# High-Frequency Plant Regeneration, Genetic Uniformity, and Flow Cytometric Analysis of Regenerants in *Ruta* *chalepensis* L.

**DOI:** 10.3390/plants10122820

**Published:** 2021-12-20

**Authors:** Ahmed A. Qahtan, Mohamad Faisal, Abdulrahman A. Alatar, Eslam M. Abdel-Salam

**Affiliations:** Department of Botany and Microbiology, College of Science, King Saud University, P.O. Box 2455, Riyadh 11451, Saudi Arabia; aqahtan@ksu.edu.sa (A.A.Q.); aalatar@ksu.edu.sa (A.A.A.); eabdelsalam@ksu.edu.sa (E.M.A.-S.)

**Keywords:** in vitro, medicinal plant, micropropagation, genetic fidelity, RAPD, DAMD

## Abstract

*Ruta chalepensis* L., an evergreen shrub in the citrus family, is well-known around the world for its essential oils and variety of bioactivities, indicating its potential medicinal applications. In this study, we investigated the effect of different culture conditions, including plant growth regulators, media types, pH of the medium, and carbon sources, on in vitro regeneration from nodal explants of *R. chalepensis*. Following 8 weeks of culture, the highest percentage of regeneration (96.3%) and maximum number of shoots (40.3 shoot/explant) with a length of 4.8 cm were obtained with Murashige and Skoog (MS) medium at pH 5.8, supplemented with 3.0% sucrose and 5.0 µM 6-Benzyladenine (BA) in combination with 1.0 µM 1-naphthaleneacetic acid (NAA). For rooting, individually harvested shootlets were transferred on ½ MS (half-strength) supplemented with IAA (indole-3-acetic acid), IBA (indole 3-butyric acid), or NAA, and the best response in terms of root induction (91.6%), number of roots (5.3), and root mean length (4.9 cm) was achieved with 0.5 µM IBA after 6 weeks. An average of 95.2 percent of healthy, in vitro regenerated plantlets survived after being transplanted into potting soil, indicating that they were effectively hardened. DNA assays (PCR-based markers) such as random amplification of polymorphic DNA (RAPD) and directed amplification of minisatellite-region (DAMD) were employed to assess in vitro cultivated *R. chalepensis* plantlets that produced a monomorphic banding pattern confirming the genetic stability. Additionally, no changes in the flow cytometric profile of ploidy between regenerated plantlets and donor plants were detected. Regeneration of this valuable medicinal plant in vitro will open up new avenues in pharmaceutical biotechnology by providing an unconventional steadfast system for mass multiplication and might be effectively used in genetic manipulation for enhanced bioactive constituents.

## 1. Introduction

*Ruta chalepensis* L. is a perennial aromatic herb belongs to Rutaceae family, known by the common name “fringed rue” and the Arabic name “Al-Shathap”. It is spread in the Mediterranean Sea area [1] and widely distributed in the Kingdom of Saudi Arabia. Nowadays, it is cultivated in many regions of the world, especially in temperate and equatorial countries [2], and usually growing on rocky slopes [3,4]. *R. Chalepensis* has various pharmacological properties, attributed to its high contents of phytochemicals such as alkaloids, flavonoids, cardiac glycosides, coumarins, tannins, saponins, anthraquinones, volatile oil, cynagenic glycosides, glucosinolates, triterpenes, sterols, amino acids, phenols, and furocoumarins [2,4,5,6,7]. In many countries, including Saudi Arabia, Yemen, Algeria, China, and India, *R. Chalepensis* is used in traditional medicine because of its biological activities, which include antioxidant, anti-bacterial [8], anti-fungal [9], anti-inflammatory [1,10], and anthelmintic [11] properties. It has been used extensively in folk medicine as an antipyretic and analgesic, as well as for the treatment of mental disorders, convulsions, rheumatism, dropsy, neuralgia, and bleeding problems [4]. Moreover, the leaves are used for treatment of epilepsy, vertigo, colic, intestinal worms, toxicity, headache, and eye problems [5]. It is used internally as an antispasmodic and antihypertensive [1,12,13].

Tissue culture has emerged as a key component of biotechnology because it allows for the propagation and mass multiplication of plants from small explants of the plant, such as segments of roots, leaves, or stems, on artificial media under controlled conditions [14,15]. This technology has played a significant role in the production of competitive and sustainable agriculture, reforestation, and biomedical applications, and has been extensively applied in breeding programs for the generation of better plants with desired traits [16,17,18,19]. Currently, it is a well-established technology for cultivating and investigating the physiological behavior of isolated plant organs, tissues, cells, protoplasts, and even cell organelles under precisely controlled chemical and physical conditions [20]. Several factors, including the selection of explant, type of medium and composition [21], plant growth regulators (PGRs), and the presence of a carbon energy source [22] that maintains osmotic potential [23], may significantly affect shoot multiplication. In tissue cultures, several different types of basal media have been formulated, and the response of different plant species to these varied media is dependent on their nutritional needs [24]. MS medium has been widely used for micropropagation purposes in a number of medicinal plants such as *Vitex negundo* [25], *Albizia lebbeck* [26] and *Centella asiatica* [27], *Artemisia sieberi* [28], *Artemisia pallens* [29], *Vitex trifolia* [30], *Ruta graveolens* [31], *Thymus persicus* [32], *Cassia alata* [33], *Bacopa monnieri* [34], and *Tecoma stans* [35,36]. WPM medium induced the highest response in *Rauvolfia tetraphylla* [37], and B5 medium had the best morphogenic response in *Saraca asoca* [24].

Plant regeneration in vitro is also influenced by the pH of the medium, which has a substantial effect on the morphogenic activity of plant tissues [25,38]. The pH of a culture medium must be within the acceptable limits, such that it does not disrupt the plant tissue [38]. The pH influences gelling efficiency of agar, where pH above 6 makes the medium very hard, and the medium does not solidify properly at less than 5 pH [39]. Appropriate pH governs several processes such as concentration of salts, uptake of PGRs, and chemical reactions, especially those catalyzed by enzymes [38]. Several studies were conducted by many researchers where a wide range of pH levels (5.5–6) was successfully tested for in vitro propagation of different plant species [25,26,40,41,42,43].

The type and concentration of carbon sources supplied to the medium as the energy source which maintain the osmotic potential are essential for efficient micropropagation [44]. Carbon sources are unavoidable as they have a partial effect on growth and morphogenesis because of their nutritional value, which impacts the rate of cell division or the level of morphogenesis of the cells [45]. Furthermore, carbon sources have an important role in the synthetic pathway of many compounds, acting as building blocks of macromolecules, and may control many developmental processes in the cell [46,47]. Carbon sources and the amount of carbohydrates act together to determine the amount of sucrose hydrolysis and the medium pH after autoclaving [48]. Hence, sugars are of prime importance for in vitro morphogenesis, a process requiring high energy [49]. Different sugars were used as a carbon source in tissue culture, such as monosaccharides (fructose, glucose, and galactose), disaccharides (sucrose, lactose, and maltose) and trisaccharide (raffinose). Meanwhile, sucrose is the most effective and promising carbon source since it is the most frequent carbohydrate found in the phloem sap of many plants [50,51], as well as because it is inexpensive, readily available, and highly efficient [52]. It has been used in the tissue culture of a number of plant species, such as *Vitex negundo* [25], *Rauvolfia serpentina* [53], *Psidium guajava* [54], *Artemisia abrotanum* [55], *Ruta graveolens* [18], *Bacopa monnieri* [34], and *Tecoma stans* [35]. Sucrose at 3% was the most effective compared to glucose, fructose, and maltose for micropropagation of *Harpagophytum procumbens* [56].

To ensure clonal stability, it is essential to evaluate the genetic integrity study between in vitro propagating plants and mother plants. In vitro culture of plants may lead to the development of somaclonal difference as a result of exposure to certain stresses during culture conditions, such as the type of PGRs used, the regeneration pathway, long-term cultures, and a large number of sub-cultures, all of which have the potential to damage DNA via cytosine methylation, nucleotide substitutions (SNS), or changes in chromosome number or structure [33,57,58,59,60]. Clonal stability in plantlets can be assessed using various techniques based on morphophysiological, biochemical, and molecular attributes. Polymerase chain reaction (PCR)-based approaches such as amplified fragment length polymorphism (AFLP), random amplified polymorphic DNA (RAPD), inter simple sequence repeats (ISSR), directed amplification of minisatellite DNA (DAMD), and simple sequence repeats (SSR) molecular markers have been considered to be quite suitable because they are reliable, easily detectable, cost-effective, do not require any prior nucleotide sequence information, and are not affected by environmental factors [60]. RAPD and DAMD marker techniques have been successfully used to assess the genetic fidelity in several medicinal plants such as *Aconitum violaceum* [61], *Gloriosa superba* [57], *Withania somnifera* [62], *Artemisia nilagirica* [63], *Cassia alata* [33], *Zanthoxylum armatum* [64], *Ruta graveolens* [18], *Bacopa monnieri* [34], *Rauvolfia tetraphylla* [59,65], *Hildegardia populifolia* [66], and *Thalictrum foliolosum* [67]. Homogeneity of in vitro propagated plants can also be ascertained using flow cytometry, which offers a quick, accurate, and simple method for assessing the ploidy level, genome size, cell cycle, and DNA content within plant nucleus homogenates [60,68]. This technique has been successfully employed to determine the nuclear DNA content, genome size, and ploidy level in *Mentha arvensis* [69], *Pongamia pinnata* [70], *Nardostachys jatamansi* [71], *Bacopa monnieri* [34], *Curcuma zedoaria* [72], and *Lippia lacunosa* [73]. 

Micropropagation studies have been reported for another species of *Ruta* genus, i.e., *R. graveolens* [18,31,74,75,76,77]. Nevertheless, no in vitro propagation studies of *R. chalepensis* have been reported. The purpose of this work was to design a successful strategy for producing *R. chalepensis* on a large scale from nodal explants by optimizing different attributes of micropropagation processes. The aptitude of the in vitro plants to survive in the ex vitro environment, as well as their genetic fidelity, were also assessed using DNA-based molecular markers (RAPD and DAMD) and flow cytometry to ensure the supply of authentic planting materials.

## 2. Results

### 2.1. Effect of Cytokinins

The morphogenetic response of *R*. *chalepensis* nodal explants to various cytokinins (BA or Kin) was evaluated (Table 1). Nodal explants cultured on MS basal medium failed to respond even after 4 weeks of culture, but MS media supplemented with varying doses of the cytokinins were able to stimulate multiple shoot induction. Analyzing the data (Table 1), the percentage of shoot induction depends on the concentration of cytokinins, and the best results were obtained in the medium containing 5.0 μM BA. After 8 weeks of incubation in this medium, 85.8% of the explants generated shoots, with an average number of 23.4 shoots/explant and an average shoot length of 4.3 cm. In contrast, medium containing 5 μM of Kin produced 13.6 shoots per explant with an average shoot length of 3.8 cm in 75.2% culture, after 8 weeks of incubation.

### 2.2. Effect of Auxins and Cytokinins

**Figure 1:**
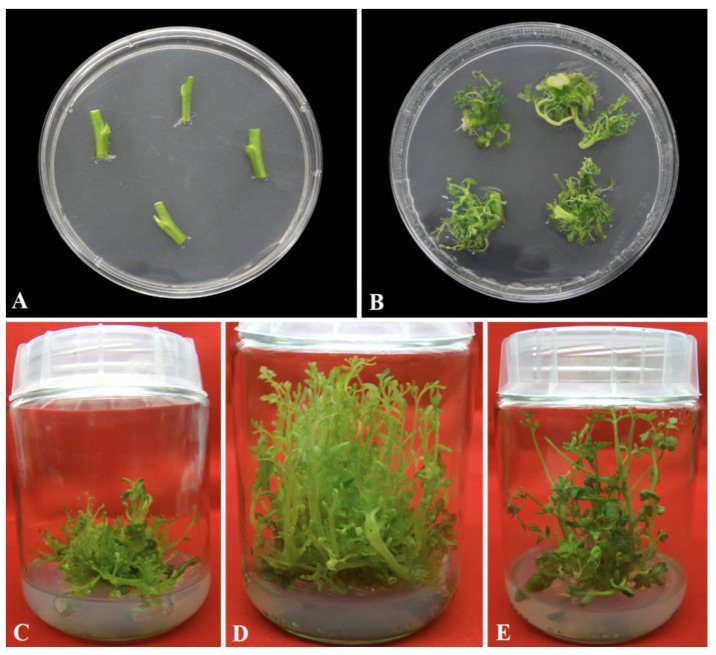
In vitro regeneration from nodal explants of *R. chalepensis.* (**A**) Explants cultured on nutrient media. (**B**) Shoot induction on MS + BA (5.0 µM) + NAA (1.0 µM) after 1 week of culture. (**C**) Four-week-old culture on MS + BA (5.0 µM) + NAA (1.0 µM). (**D**) Proliferated shoots on MS + BA (5.0 µM) + NAA (1.0 µM) after 8 weeks of culture. (**E**) Shoot induction on MS + Kin (5.0 µM) + NAA (1.0 µM) after 8 weeks of culture.

The synergistic effect of auxins (IAA, IBA, and NAA) at various concentrations (0.5, 1.0, 2.0, and 2.5 μM) with an optimal concentration (5.0 μM) of BA or Kin was tested on multiple shoot inductions. The obtained results were compared with 5.0 µM of BA as a control. Shoot induction started after one week of incubation in all examined media. When compared to other auxins investigated, NAA was shown to be the most potent, followed by IAA and IBA. NAA (1.0 µM) in combination with BA (5.0 µM) showed the highest response (96.33% of cultures after 8 weeks) among all other treatments. Furthermore, the combination showed the largest number of shoots (40.3 per explant) with an average shoot length of 4.8 cm after 8 weeks. However, an equimolar dose of BA with IAA produced 32.6 shoots/explant with an average shoot length of 4.6 cm in 90.0% cultures after 8 weeks (Figure 1). A similar dose of BA with IBA produced the lowest number of shoots (26.2 per explant) with an average shoot length of 3.1 cm and 86.3% regeneration frequency after 8 weeks of culture.

A combination of NAA (1.0 μM) and Kin (5.0 μM) in MS media led to 25.6 shoots/explant with an average shoot length of 4.9 cm in 90.3% cultures after 8 weeks (Table 2; Figure 1). However, presence of IAA or IBA (0.5–2.5 µM) with Kin (5.0 µM) was less effective than NAA for shoot induction. The maximum number of shoots per explant was found at 1.0 µM IAA or IBA, with 19.6 and 17.8 shoots per explant, respectively.

### 2.3. Effect of Different Medium and pH Levels

**Figure 2:**
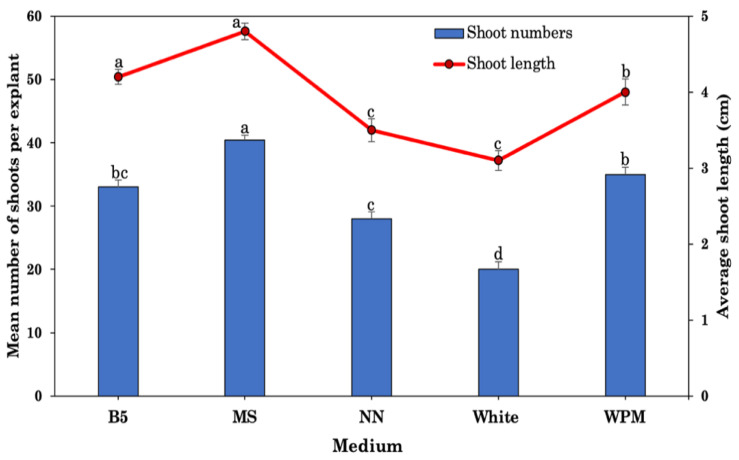
Effects of different types of media with 5.0 BA and 1.0 μM NAA on shoot regeneration in *R. chalepensis*. B5: Gamborg’s medium; MS: Murashige and Skoog medium; WPM: Woody plant medium; White: White’s medium; NN: Nitsch and Nitsch medium. Bars without a common letter differ (*p* ≤ 0.05), as analyzed by one-way ANOVA and Duncan’s multiple range test.

In this study, different tissue culture media, such as MS, B5, WPM, NN, or White’s, were investigated for their ability to promote shoot multiplication and elongation when supplemented with the optimal concentration and combination of PGRs (5.0 μM BA and 1.0 μM NAA). MS media was found to be the best for shoot induction and multiplication in *R. chalepensis*, resulting in the largest number of 40.3 shoots per explant and the longest average shoot length of 4.8 cm after 8 weeks of culture (Figure 2). Compared to the other media investigated in this research, White’s basal medium produced the fewest shoots.

**Figure 3:**
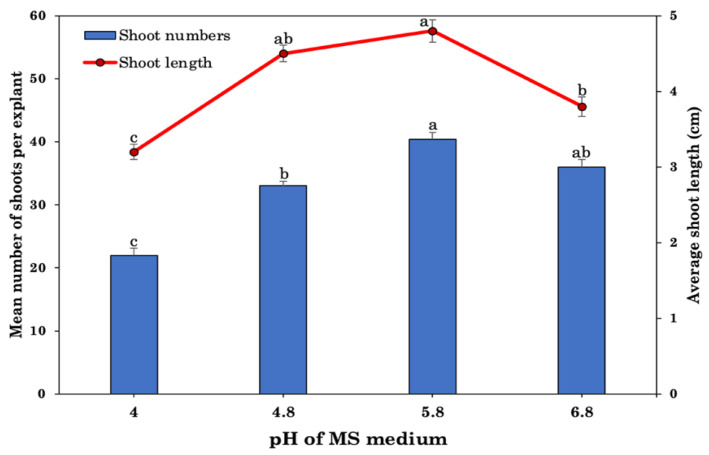
Effects of different pH levels of media with 5.0 BA and 1.0 μM NAA on shoot regeneration from nodal explants of *R. chalepensis*. Bars without a common letter differ (*p* ≤ 0.05), as analyzed by one-way ANOVA and Duncan’s multiple range test.

Effect of varied pH levels (4, 4.8, 5.8, and 6.8) of the MS medium on induction and multiplication shoots in *R. chalepensis* were investigated with optimum 5.0 BA and NAA concentrations. The pH level of the MS medium exhibited a differential response, and after 8 weeks of culture, the pH of 5.8 resulted in the highest number of shoots per explant with the longest shoot length (Figure 3). As a result, the pH value of 5.8 was shown to be optimal for maximal shoot regeneration and multiplication. The number of shoots decreased when the pH level was raised to 6.8. Furthermore, increasing the acidity of the medium (pH = 4.0) resulted in a reduction in shoot induction and proliferation.

### 2.4. Effects of Carbon Sources

**Figure 4:**
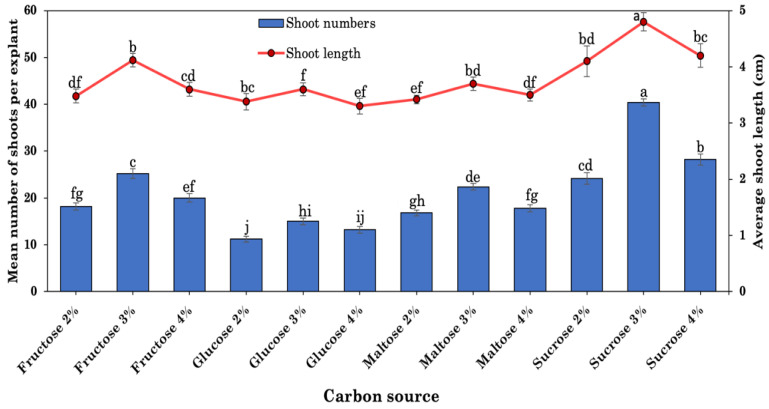
Effects of carbon source on shoot regeneration from nodal explants of *R. chalepensis* cultured on MS medium supplemented with 5.0 BA and 1.0 μM NAA. Bars without a common letter differ (*p* ≤ 0.05), as analyzed by one-way ANOVA and Duncan’s multiple range test.

Glucose, sucrose, fructose, and maltose were evaluated at varied concentrations (2, 3, or 4 percent, *w/v*) in the optimized medium to determine the influence on shoot multiplication from nodal explants of *R. chalepensis*. At 3 percent, each individual carbon source had the highest response rate as well as the greatest number of shoots. After 8 weeks of culture, any decrease or increase in concentration resulted in a substantial decrease in response rate and number of shoots. After 8 weeks of culture, the highest frequency (96.3%) of shoot multiplication and maximum shoot number (40.3 shoot/explant), along with the longest shoot length 4.8 cm) were observed on MS media containing 5.0 μM BA, 1.0 μM NAA, and 3% (*w/v*) sucrose (Figure 4).

### 2.5. Rooting of Shootlets

**Figure 5:**
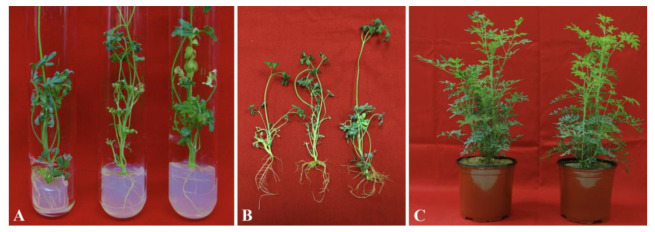
(**A**) Rooting induction from microshoots *R*. *chalepensis* on MS + IBA (0.5 μM). (**B**) Rooted shootlets before transplantation; (**C**) after 6 months of field transfer.

Elongated and healthy microshoots (≥5 cm) were obtained from 8-week-old cultures and cultivated on half-strength MS media either without or with auxins (IAA, NAA, or IBA) at varying concentrations (0.1, 0.5, 1.0, and 2.0 μM) for in vitro rhizogenesis. In most media examined, rooting induction occurred between 2 and 3 weeks from the cut ends of the microshoots (Figure 5A). Even after four weeks of culture, microshoots grown in MS media without phytohormones were unable to induce roots. After 6 weeks of incubation, the MS medium supplemented with 0.5 μM IBA had the best response in terms of root induction (91.6%), maximum number of roots/shoot (5.3), and the longest root length (4.9 cm; Table 3). Among the tested NAA concentrations, best response of root induction (90.3%) with maximum number of roots/shoot (4.3) and longest root length (2.3 cm) was recorded on 0.5 μM NAA containing media after 6 weeks of incubation. On the other hand, the incubated microshoots on MS medium supplemented with IAA produced the best response of root induction (81.3%) with maximum number of roots/shoot (2.6) and longest root length (2.1 cm) on media containing 0.5 μM IAA. The root inductions were decreased in media containing a higher concentration of 2.0 μM IBA, NAA, or IAA, with only 53.0%, 52.0%, and 43.0% of the microshoots being able to induce the roots, respectively. All NAA concentrations resulted in callus development at the end cut of microshoots. The roots obtained on IBA were healthy, elongated, and thick, leading to efficient acclimation.

### 2.6. Effect MS Basal Media Strengths on Rooting

The effect of several MS medium strengths (¼, ¾, ½, and full MS) supplemented with the optimum dose of 0.5 μM IBA on in vitro rooting was investigated. The findings revealed that ½ MS was the best for in vitro rooting since it generated even more roots/shoot (5.4) and the longest average root length (4.9 cm) after 6 weeks of incubation (Table 4).

### 2.7. Acclimatization

Healthy regenerated plantlets of *R*. *chalepensis* with 4–8 leaves and a well-developed root system (Figure 5B) were successfully hardened off in potting soil within a growth chamber prior to being transplanted ex vitro. After 6 weeks, the acclimatized plants were transferred to pots having normal garden soil, and about 95.2% of the plantlets survived when transferred to field condition. After 6 months of field transfer, the established plants were growing normally, and no changes were observed in the morphological characters between the micropropagated and donor plants (Figure 5C).

### 2.8. Assessment of Genetic Stability

**Figure 6:**
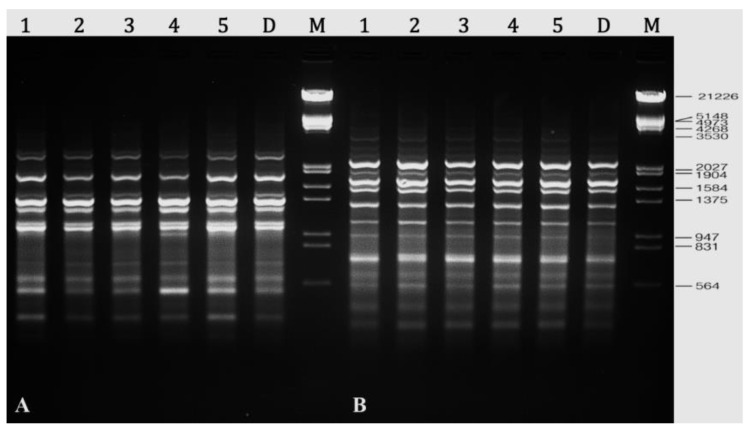
Representative RAPD profiles of *R. chalepensis*. (**A**) Primer A-01 amplified profile. (**B**) Primer A-10 amplified profile. Lanes 1–5 randomly selected in vitro plants; lane D—donor plant; lane M—DNA marker.

In this study, twelve RAPD primers were tested, and all of the primers generated bands that were clear, scorable, and repeatable. There were 107 well resolved and reproducible bands generated by these twelve primers, with an average of 8.9 bands generated by each primer (Table 5). It was revealed that primer A-10 had produced the most amplified DNA bands (15), while primer A-02 produced the fewest bands (03). RAPD primers generated monomorphic bands in all of the in vitro plants that were assessed and compared to their parent plant (Figure 6).

**Figure 7:**
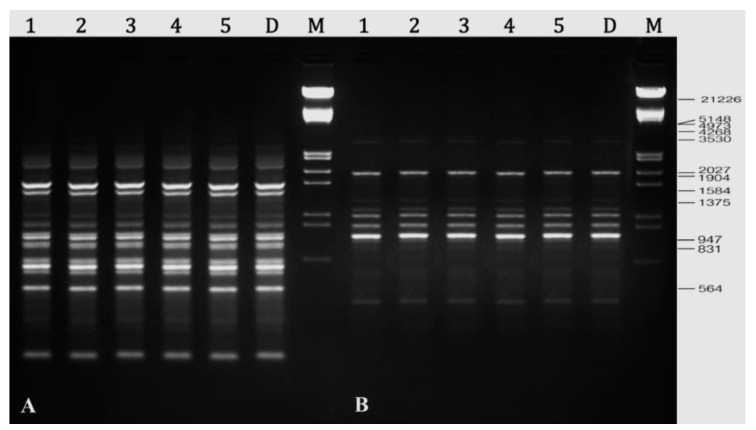
Representative DAMD profiles of *R. chalepensis*. (**A**) Primer HVR amplified profile. (**B**) Primer M13 amplified profile. Lanes 1–5 randomly selected in vitro plants; lane D—donor plant; lane M—DNA marker.

Clear and scorable bands were also produced by all five DAMD primers tested in PCR amplification. These primers produced a total of 61 bands, with an average of 12.2 bands per primer; the number of bands produced ranged from 10 (HVR and M13) to 15 (HBV5) (Table 6). The regenerated *R*. *chalepensis* plants were found to be genetically identical to donor plants with 0% polymorphism (Figure 7).

### 2.9. Flow Cytometric Analysis

**Figure 8:**
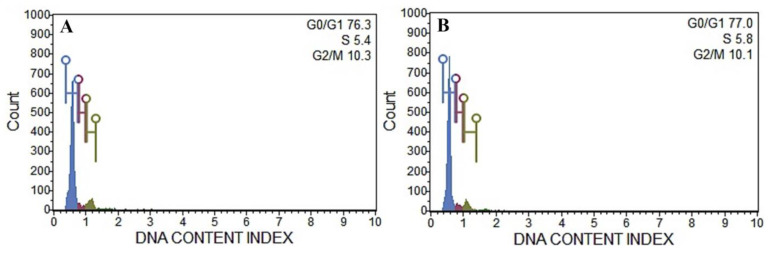
Flow cytometric histograms obtained from nuclei of *R. chalepensis* leaf samples. (**A**) Ex vitro plants; (**B**) in vitro regenerated plants.

Cell cycle analysis was performed using a flow cytometer to determine the ploidy level and DNA content index of the regenerated plants, and the results were compared to those of the donor plant. The histogram of DNA content index (nDNA) obtained from the nuclei samples of both plants is presented in the Figure 8, which showed a unimodal fluorescence peak of the nDNA content corresponding to 2×. Following the present cell cycle study, it was found that all plant sources had nearly identical G0/G1 positions in the histogram (Figure 8), indicating that there is no difference in nDNA profile and ploidy status between in vitro and ex vitro cultivated *R. chalepensis* plants (Figure 8).

## 3. Discussion

PGRs play a crucial role in in vitro morphogenesis such as callus induction, shoot regeneration, and somatic embryogenesis, and the levels of PGRs needed in plant tissue culture may differ across species. According to the findings of this investigation, nodal explants of *R. chalepensis* that were cultivated in a nutrient-rich medium supplemented with a variety of BA or Kin concentrations (1–10 M) demonstrated a variable response when exposed to different concentrations of BA or Kin. The frequency of shoot regeneration from node explants was the highest in MS medium containing (5.0 µM) BA. The type of exogenously administered cytokinins, as well as their concentration, absorption, and transport, have an effect on the success of the in vitro propagation method [78,79]. The effect of BA on shoot induction and multiplication from node explants has been reported by many authors [67,74,80,81,82,83,84]. The effect of optimized level (5.0 µM) of BA with lower concentration (1.0 µM) of auxins (NAA) produced a better response in terms of greatest shoot induction and proliferation from nodal explant of *R. chalepensis*. The findings of this study demonstrate that a proper balance of cytokinin and auxin (BA/NAA) is required for optimum shoot regeneration. When a relatively high ratio of cytokinins is used in addition to the low ratio of auxins, it affects cell division, promoting a higher frequency of shoot bud induction and inducing a greater number of shoots/explants over cytokinin alone [85,86]. The positive effects of auxin additions on regeneration rates could be attributable to the potential synergistic effects between auxins and cytokinins that enhance the physiological response of regenerated plants and promote their development and proliferation [87]. Furthermore, the combined action of cytokinins and auxins has an important role in cell division and multiplication to form new cells, each of which appears to influence different phases of the cell cycle. In addition to having an influence on DNA replication, cytokinin seems to have some control over the processes leading up to mitosis. Therefore, using cytokinin and auxin in combination increases the efficacy of culture media for maximal shoot multiplication [88]. Based on our findings, nodal explants *R. chalepensis* cultivated on MS basal medium with NAA (1.0 µM) and BA (5.0 µM) exhibited the highest response percentage (96.3%) and the maximum number of shoots (40.3 per explant) when compared to other treatments. These findings are consistent with previous studies in which the highest levels of shoot proliferation were observed in a wide range of plant species, including *Ruta graveolens* [18,74,76], *Syzygium cumini* [80], *Hildegardia populifolia* [66], and *Mondia whitei* [82].

Plant regeneration success is contingent upon the kind of nutritional medium employed, as well as its composition, pH, and carbon supply. To maximize differentiation and growth of explants, the culture medium’s mineral salts, organic additions, and pH of the media must be carefully balanced. However, plant species usually differ in their requirements, and therefore, they respond differently to different basal media. The pH of medium may be ascribed to medium components, autoclaving, ion exchange, and ambient conditions, among other factors. The pH of the medium may be altered both before and after autoclaving depending on the medium components [89,90]. For in vitro shoot regeneration and tissue culture, MS media with a pH of 5.8 has been shown in a number of studies to be the most appropriate medium [34,44,53]. Shoot regeneration from explants cultivated in MS medium with a pH of 5.8 was determined to be optimal in this study, according to the findings. However, shoot differentiation and growth were found less effective in B5, WPM, NN, and White media with a pH 5.8. MS medium with a pH 5.8 has also been found significantly more effective than other media in various plants species, e.g., *Albizia lebbeck* [26], *Vitex negundo* [25], *Centella asiatica* [27], *Plumbago zeylanica* [91], and *Physocarpus opulifolius* [92].

The pH of the medium may be influenced by a variety of events, including hydrolysis, enzyme breakdown, photooxidation, and photolysis on light-sensitive medium components. Water splitting and glycosidic bond breaking are frequent requirements for sucrose hydrolysis. Because tissue culture media is frequently adjusted to slightly acidic conditions (5.8), autoclaving offers a temperature that is adequate for catalyzing sucrose hydrolysis. It has been shown that acid-facilitated autocatalyzed sucrose hydrolysis is both pH- and temperature-dependent, with lower pH at a given temperature promoting greater sucrose hydrolysis [93,94]. Carbon sources and the amount of carbohydrates act together to determine the amount of sucrose hydrolysis and the medium pH after autoclaving [48]. Carbohydrates such as sucrose, glucose, fructose, and maltose are often used in the establishment of plant tissue culture. Sucrose is broadly applied in in vitro culture because of its positive effects on plant growth and development and low cost. Successful in vitro organogenesis, on the other hand, is highly dependent on the concentration of sucrose used and its interaction with other medium compositions. In the current study, the effects of different types of carbohydrates, i.e., sucrose, glucose, fructose, and maltose, at various concentrations were tested. Sucrose was shown to be the most effective sugar for promoting shoot regeneration from nodal explants in this study, followed by fructose, maltose, and glucose. Similar results were obtained in micropropagation of *Harpagophytum procumbens* [56], *Aquilaria malaccensis* [95], *Plumbago zeylanica* [91], *Pterocarpus marsupium* [96], and *Lupinus albus* [97]. Sucrose, the most prevalent carbohydrate in plant phloem sap [50,51], is often used in tissue culture due to its inexpensive cost, easy availability, and ability to quickly absorb through the plasma membrane [74], which aids in sugar hydrolysis as well as maintain the pH of the medium [48].

One of the most essential aspects in the micropropagation process is the development of a robust root system. In this study, microshoot rooting (5.4 roots per microshoot) was best obtained in ½ MS basal medium with 0.5 M of IBA. Similar results were reported in *Ruta graveolens* [18,74], *Rauvolfia serpentina* [53], *Hemidesmus indicus* [98], *Maerua oblongifolia* [99], *Rauvolfia tetraphylla* [59], *Artemisia vulgaris* [100], *Thalictrum foliolosum* [67], and *Asystasia gangetica* [101]. In a wide range of plant species, IBA was shown to be the most suited for in vitro root induction over IAA or NAA because of its greater resilience to photodegradation, adsorption to microshoots, and deactivation with biological action [102]. IBA is also quickly absorbed, retained, and transported throughout plant tissues, and it has the potential to activate the gene responsible for rhizogenesis [103]. IAA and NAA were shown to be less efficient in inducing roots than IBA, owing to the fact that IAA is readily oxidized by light and denatures rapidly in culture media, but NAA may survive longer in plant tissues [88].

The development of somaclonal anomalies between the regenerated plants may limit the effectiveness of the micropropagation protocol [33,104]. Explant preparation and subsequent sub-culturing of sub-clones for extended durations in tissue culture systems might lead to these changes [105,106,107]. Because of this, evaluating the genetic integrity of micropropagated plants is critical. PCR-based markers are one of the most significant approaches being used test genetic stability in several plant species, since it is not affected by culture conditions and may be acquired at any stage of plant development. ISSR, RAPD, AFLP, SSR, and DAMA are the most frequently employed techniques since they do not need any previous DNA sequence information [33,106]. In the present investigation, RAPD and DAMD markers were applied to assess the genetic homogeneity of *R*. *chalepensis* plantlets. All RAPD and DAMD-generated bands were monomorphic and identical, confirming the complete absence of somaclonal variations in regenerated plantlets. It has been shown that the use of RAPD and DAMD molecular markers is efficient in determining the genetic stability of regenerated plants in a variety of medicinal plant species, including *Withania somnifera* [108], *Henckelia incana* [109], *Avicennia marina* [110], *Ruta graveolens* [18], *Curcuma zedoaria* [72], *Artemisia vulgaris* [100], *Anarrhinum pubescens* [111], and *Anthurium andraeanum* [106]. In recent years, it has been shown that a flow cytometry-based approach for evaluating in vitro raised plantlets is an excellent technique for assessing the clonal integrity and ploidy status in micropropagated plants [34,71,72,112,113]. In the present investigation, no major differences between fluorescence peak derived from nuclei of *Ruta chalepensis* plantlets and ex vitro plants were found. The findings of flow cytometric analysis are consistent with those obtained from earlier research on *Mentha arvensis* [69], *Puya berteroniana* [112], *Solanum lycopersicum* [113], *Cucumis melo* [114], *Bacopa monnieri* [34], *Curcuma zedoaria* [72], and *Juglans regia* [115].

## 4. Materials and Methods

### 4.1. Plant Material and Surface Sterilization

The young, healthy stem segments of *R. chalepensis* were harvested from a plant growing in the Botany and Microbiology Department of the King Saud University in Riyadh, Saudi Arabia. The explants were washed for 25 min in laboratory tap water, then treated for 5 min in a 5% (*v/v*) liquid detergent, followed by 4–5 rinses with sterile ultrapure (Milli-Q) water to remove any detergent residue. Inside a laminar air flow hood (ESCO Labculture^®^ Class II Type A2 Biological Safety Cabinet, Esco Micro Pte. Ltd., Singapore), the plant materials were surface sterilized with 0.1 percent (*w/v*) mercuric chloride (HgCl_2_) for 3 min at room temperature. The HgCl_2_-treated explants were finally rinsed 4–5 times with sterile ultrapure (Milli-Q) water and cut into pieces that were about 0.5–0.7 cm in diameter, which were then utilized for further in vitro assays.

### 4.2. Preparation of Media and Culture Conditions

The sterile nodal segment explants of *R. chalepensis* were aseptically cultured onto Murashige and Skoog (MS) [116] agar medium with 3% (*w/v*) sucrose and various combinations and concentrations of growth regulators (auxins and cytokinins), as specified below. pH of the nutrient media was adjusted to 5.8 by the addition of 1 M NaOH or HCl before adding 0.8% (*w/v*) agar. The media was then autoclaved at 121 °C and 15 psi for 20 min. The media was dispensed into petri dishes, each of which contained 25 mL of medium. Following the inoculation of the explants, the dishes were sealed with one layer of parafilm. All cultured plates/vials were maintained at a temperature of 24 ± 2 °C, a photoperiod of 16/8 h (day/night), a photon flux density of 50 mol m^−2^ s^−1^ provided by cool LED tubes, and a relative humidity of 50–60%.

### 4.3. In vitro Shoot Initiation and Proliferation

For shoot initiation and multiplication, the nodal explants of *R. chalepensis* were cultured on MS medium supplied with varying concentrations of cytokinins, i.e., 6-benzyladenine (BA) or Kinetin (Kin) (1.0, 2.5, 5.0, 7.5, or 10 μM), individually or in combination with auxins, i.e., indole-3-acetic acid (IAA), 1-naphthaleneacetic acid (NAA), and indole 3-butyric acid (IBA), at various concentrations (0.5, 1.0, 1.5, or 2.0 μM). All cultures were subcultured in the same fresh medium every three weeks.

### 4.4. Effect of Various Media and pH

The use of the optimal growth regulators and a suitable media in in vitro culture is essential for overall growth response of the explants. In order to find the best basal media for shoot initiation and development from nodal explants of *R. chalepensis*, we examined MS medium, Gamborg’s Medium (B5) [117], Woody Plant Medium (WPM) [118], White’s medium [119], and Nitsch and Nitsch (NN) Medium [120]. Each basal medium comprised 3% sucrose, 0.8% agar, and the optimized auxin and cytokinin (5.0 μM BA and 1.0 μM NAA) combination. Effect of different pH levels (4, 4.8, 5.8, and 6.8) of the optimized nutrient medium on shoot development were also evaluated. After three weeks, all the responding explants with shoot clumps were sub-cultured on the fresh culture medium, and the data on the number of shoots and the length of the shoots were collected after eight weeks of cultivation.

### 4.5. Effects Carbon Sources

Response of explants to several carbon sources, such as sucrose, glucose, maltose, and fructose, at concentrations of 2, 3, and 4 percent (*w/v*), were assessed in MS media augmented with 5.0 μM BA, 1.0 μM NAA. Following 8 weeks of culture, the frequency with which *R. chalepensis* explants produced shoots, number of shoots per explant, and the shoot length were determined.

### 4.6. Rooting of Shootlets

Rooting of the regenerated microshoots of *R. chalepensis* was accomplished by the use of an in vitro rooting approach. Isolated healthy microshoots (4–5 cm) were transplanted to culture tubes with half-strength MS without PGRs (as a control) or supplemented with 0.1, 0.5, 1.0, or 2.0 μM of an auxin such as IAA, IBA, or NAA. To find the optimal medium for root induction, different strengths of MS nutrient agar media (¼, ¾, and full strength) coupled with the better dose of an auxin (0.5 μM IBA) were examined.

### 4.7. Acclimatization

Plantlets with a well-developed shoot and root systems were carefully removed from the culture tube and rinsed with normal water to eliminate any remaining agar. A high level of humidity was maintained around the plant by implanting plantlets in pots containing potting soil (Planta Guard, Germany) and covering the pots with transparent plastic covers. The plantlets were kept in a growth environment under a 16/8 h (day/night) photoperiod with a photon flux density of 50 mol m^−2^ s^−1^, high humidity (50–60 percent), and watered with ¼ MS devoid of organic nutrients every three days for three weeks before being irrigated with regular water. The coverings were perforated and progressively removed over a period of 12–20 days in order to allow the plantlets to become more acclimated to field conditions. After 6 weeks, these well-acclimated plantlets were transplanted into pots filled with standard garden soil and kept in a greenhouse under natural day light conditions.

### 4.8. Flow Cytometric Analysis

Flow cytometric analysis portable Muse Cell Analyzer (Muse^®^ Cell Analyzer, Merck Millipore, USA) was used to compare the ploidy level of *R*. *chalepensis* donor and in vitro regenerated plants. Using a sharp scalpel blade (No. 21), approximately 100 mg of fresh leaf samples was chopped in 1 mL of Galbraith buffer (pH 7.0) containing 0.1% (*v/v*) Triton X-100, 20 mM MOPS, 30 mM sodium citrate, and 45 mM MgCl_2_ [68] to isolate the nuclei. The isolated nuclei in buffer were filtered through double-layered 28 µm nylon meshes, and then, 50 µg/mL of propidium iodide (PI, Sigma, USA) solution was added and mixed for 15 min. Finally, 50 µg/mL RNase (Sigma, USA) were added to avoid staining of double-stranded RNA molecules and the nuclei samples were passed through the Muse Cell Analyzer. Each run had at least 5000 nuclei, and each experiment was repeated three times.

### 4.9. DNA Extraction and PCR Amplification

Young leaf samples were collected from the donor plant and randomly selected micropropagated plants. DNA was extracted from leaf samples (approximately 250 mg) by the cetyltrimethylammonium bromide (CTAB) method [121]. DNA purity and concentration were determined using a Nanodrop spectrophotometer (Nanodrop 2000, Thermo Scientific, USA). The purity and quality of the DNA were also checked using 1% agarose gel (1X TBE buffer) stained with ethidium bromide using gel electrophoresis. DNA samples were diluted to a final concentration of 25 ng/μL in DNAse, RNAse free ultrapure water (Milli QR, Millipore, USA) for PCR reactions. Twelve RAPD (GeneLink, Inc., Orlando, FL, USA) and five DAMD (GeneLink, Inc., Orlando, FL, USA) primers were used for screening of DNA amplification. Then, 20 µL of PCR reaction comprising 10 µL of PCR master mix (GoTaq^®^ Green Master Mix, 2X, Promega, USA), 1 µL genomic DNA (50 ng/μL), 1 µL primer, and 8.0 µL ultrapure (Milli-Q) water. Thermal Cycler, Bio-Rad, USA) was used to perform the PCR reactions, which were configured to complete 40 cycles after an initial denaturation cycle of 5 min at 94 °C. Each cycle included a denaturation step at 94 °C for one minute, an annealing step at 29.5–57 °C for one and a half minutes, an extension step at 72 °C for two minutes, and a final extension cycle at 72 °C for seven minutes. All of the experiments were repeated three times in order to avoid false outcomes and to validate the reproducibility of the RAPD and DAMD markers. The PCR products were separated by electrophoresis on 1.5 percent agarose gels with 5 µL ethidium bromide in 1X Tris-borate-EDTA (TBE) buffer at 75 V for 2 h and photographed using the gel documentation system (G:BOX F3, Syngene, Cambridge, UK).

### 4.10. Statistical Analysis

IBM-SPSS software for Macintosh version 26.0 (IBM-SPSS Inc., IL, Chicago, USA) was used for statistical data analysis in one of the experiments, which had a totally randomized design with 20 replicates and was performed three times. The collected data were subjected to analysis of variance (ANOVA), and Duncan’s multiple range tests were used to evaluate the significant differences (*p* ≤ 0.05) between the treatment values.

## 5. Conclusions

In conclusion, we developed an excellent approach for direct in vitro regeneration of *R. chalepensis*—a valuable medicinal plant—by evaluating several parameters such as PGRs and their combinations, media types, media pH, and carbon source. According to a data analysis of all parameters, the best response in terms of shoot regeneration was achieved in MS medium at pH 5.8 supplemented with 3% sucrose and 5 µM BA in combination with 1 µM NAA. The method that was devised should enable for the mass multiplication of these multipurpose plants, the development of large-scale nurseries for ex situ conservation, as well as the commercial exploitation of these plants in the biopharmaceutical industry. Flow cytometry and DNA-based markers, such as RAPD and DAMD, were utilized to validate that the plants grown in vitro were true to type, ensuring the delivery of authentic planting materials. The high-frequency regeneration of this potential medicinal plant not only provides an alternative viable system for rapid clonal multiplication, but also opens up new avenues in pharmaceutical biotechnology by providing an unconventional steadfast system for commercial interests, and it may be successfully used in genetic manipulation for enhanced secondary metabolites and essential oil.

## Figures and Tables

**Table 1:** Effects of different concentrations of cytokinins, i.e., 6-benzylaminopurine (BA) and kinetin (Kin), on shoot multiplication from nodal explants *R. chalepensis* after 8 weeks of culture.

Cytokinins (µM)		Response%	Mean no. of Shoots	Mean Shoot Length (cm)
BA	Kin			
0	0	0 ± 0 ^f^	0 ± 0 ^e^	0 ± 0 ^i^
1.0		68.43 ± 1.64 ^f^	16.45 ± 1 ^c^	2.92 ± 0.11 ^d^
2.5		77.5 ± 0.92 ^b,c^	19.14 ± 0.71 ^b^	3.16 ± 0.16 ^d^
5.0		85.82 ± 0.72 ^a^	23.42 ± 0.81 ^a^	4.38 ± 0.29 ^a^
7.5		78.44 ± 0.98 ^b^	19.81 ± 0.86 ^b^	4.1 ± 0.1 ^b^
10		73.83 ± 1.1 ^d,e^	18.04 ± 0.72 ^b,c^	3.76 ± 0.18 ^c^
	1.0	55.47 ± 0.89 ^h^	10.26 ± 0.8 ^e^	2.78 ± 0.1 ^d^
	2.5	65.91 ± 0.84 ^g^	10.72 ± 15 ^e^	2.96 ± 0.15 ^d^
	5.0	75.25 ± 0.79 ^c,d^	13.6 ± 0.4 ^d^	3.88 ± 0.17 ^c^
	7.5	72.28 ± 0.75 ^e^	10.42 ± 0.51 ^e^	2.92 ± 0.05 ^d^
	10	69.01 ± 0.66 ^f^	9.6 ± 0.81 ^e^	2.86 ± 0.05 ^d^

Values are means ± SEM, n = 20 per treatment group. Means in a row without a common superscript letter differ (*p* ≤ 0.05), as analyzed by one-way ANOVA and Duncan’s multiple range test.

**Table 2:** Effects of different concentrations of auxins, i.e., 1-naphthaleneacetic acid (NAA), indole-3-acetic acid (IAA), and indole 3-butyric acid (IBA), with optimal concentrations of 6-benzylaminopurine (BA; 5.0 μM) and kinetin (Kin; 5.0 μM) on shoot induction from nodal explants *R. chalepensis* after 8 weeks of culture.

Cytokinins (µM)		Auxins (µM)			Response %	Mean no. of Shoots	Mean Shoot Length (cm)
BA	Kin	NAA	IAA	IBA			
5.0	-	-	-	-	85.82 ± 0.72 ^c^	23.42 ± 0.81 ^f^	4.38 ± 0.29 ^b,d^
5.0	-	0.5	-	-	80.71 ± 2.34 ^e^	25.43 ± 1.2 ^d,e^	3.78 ± 0.15 ^e,f,g^
5.0	-	1.0	-	-	96.33 ± 2.02 ^a^	40.37 ± 1.45 ^a^	4.8 ± 0.15 ^a,b^
5.0	-	1.5	-	-	83.44 ± 1.67 ^d^	30.44 ± 1.4 ^c^	4.32 ± 0.17 ^b,d^
5.0	-	2.0	-	-	70.02 ± 1.73 ^k^	21.12 ± 1.16 ^g,h^	3.28 ± 0.17 ^g,h,i,j^
5.0	-	-	0.5	-	79.36 ± 1.76 ^e,g^	22.7 ± 1.20 ^i,j,k^	3.34 ± 0.15 ^g,h,i,j^
5.0	-	-	1.0	-	90.04 ± 2.45 ^b^	32.66 ± 1.46 ^b^	4.66 ± 0.09 ^a,b^
5.0	-	-	1.5	-	80.11 ± 2.33 ^e,f^	25.41 ± 1.1 ^d^	4.3 ± 0.16 ^b,d^
5.0	-	-	2.0	-	70.05 ± 2.35 ^k^	20.31 ± 18 ^g,i^	3.12 ± 0.19 ^h,i,l^
5.0	-	-	-	0.5	75.16 ± 0.64 ^i^	20.63 ± 0.51 ^g,i^	2.76 ± 0.08 ^l^
5.0	-	-	-	1.0	86.37 ± 0.66 ^c^	26.23 ± 0.58 ^d^	3.16 ± 0.14 ^h,i,l^
5.0	-	-	-	1.5	78.51 ± 0.79 ^f,g^	22.24 ± 0.86 ^f g^	2.92 ± 0.11 ^i,l^
5.0	-	-	-	2.0	66.4 ± 0.76 ^m^	18.87 ± 0.86 ^i,j,k^	2.78 ± 0.06 ^k,i^
-	5.0	0.5	-	-	78.62 ± 0.86 ^f,g^	16.4 ± 0.68 ^l,m,n^	3.42 ± 0.23 ^f,i^
-	5.0	1.0	-	-	90.31 ± 0.36 ^b^	25.61 ± 18 ^b^	4.9 ± 0.25 ^a^
-	5.0	1.5	-	-	80.23 ± 0.51 ^e,f^	20.43 ± 0.87 ^g,i^	3.78 ± 0.18 ^e,f,g^
-	5.0	2.0	-	-	68.32 ± 0.5 ^i^	14.21 ± 1.28 ^o^	2.84 ± 0.23 ^j,l^
-	5.0	-	0.5	-	76.44 ± 0.63 ^h,i^	14.82 ± 0.66 ^m,o^	2.84 ± 0.23 ^j,l^
-	5.0	-	1.0	-	86.01 ± 0.56 ^c^	19.6 ± 0.51 ^h,i,j^	4.18 ± 0.22 ^c,d,e^
-	5.0	-	1.5	-	77.74 ± 0.74 ^g,h^	17.26 ± 0.37 ^k,l^	3.62 ± 0.14 ^f,h^
-	5.0	-	2.0	-	67.55 ± 0.6 ^l,m^	14.02 ± 0.71 ^o^	3.16 ± 0.14 ^h,i,l^
-	5.0	-	-	0.5	71.22 ± 0.65 ^j,k^	13.4 ± 0.51 ^o^	2.72 ± 0.19 ^l^
-	5.0	-	-	1.0	80.6 ± 0.56 ^e^	17.82 ± 0.74 ^j,l^	3.88 ± 0.12 ^d,f^
-	5.0	-	-	1.5	72.74 ± 0.67 ^i^	16.45 ± 0.93 ^l,m,n^	3.18 ± 0.42 ^e,f,g^
-	5.0	-	-	2.0	64.67 ± 0.96 ^n^	14.47 ± 0.93 ^n,o^	3.04 ± 0.15 ^i,l^

Values are means ± SEM, *n* = 20 per treatment group. Means in a row without a common superscript letter differ (*p* ≤ 0.05) as analyzed by one-way ANOVA and Duncan’s multiple range test.

**Table 3:** Effects of different auxins on rooting from regenerated shoots of *R*. *chalepensis* after 6 weeks of incubation.

Auxins (μM)			Response%	No. of Roots/Microshoots	Root Length (cm)
IAA	NAA	IBA			
0	0	0	0 ± 0 ^f^	0 ± 0 ^f^	0 ± 0 ^f^
0.1			57.61 ± 1.45 ^d^	1.4 ± 0.3 ^f,g^	1.54 ± 0.11 ^e^
0.5			81.33 ± 2.03 ^b^	2.6 ± 0.33 ^c.d^	2.14 ± 0.11 ^c,d,e^
1.0			70.4 ± 1.33 ^c^	1.9 ± 0.19 ^d,e,f^	1.82 ± 06 ^d,e^
2.0			43.02 ± 1.67 ^f^	1.19 ± 0.17 ^g^	1.56 ± 0.16 ^e^
	0.1		704 ± 1.15 ^c^	2.51 ± 0.34 ^c.d.e^	1.98 ± 0.37 ^b,c^
	0.5		90.31 ± 2.15 ^a^	4.34 ± 0.33 ^a,b^	2.3 ± 0.13 ^b,c^
	1.0		702 ± 1.45 ^c^	2.32 ± 0.31 ^c,d,e^	2.04 ± 0.14 ^b,c^
	2.0		52.04 ± 2.01 ^e^	1.55 ± 0.28 ^e,f,g^	1.90 ± 0.15 ^c^
		0.1	711 ± 2.33 ^c^	4.04 ± 0.57 ^b^	2.96 ± 0.69 ^b,c^
		0.5	91.63 ± 2.88 ^a^	5.38 ± 0.51 ^a^	4.91 ± 0.39 ^a^
		1.0	71.36 ± 2.64 ^c^	3.34 ± 0.34 ^b,c^	3.27 ± 0.69 ^b^
		2.0	53.01 ± 1.85 ^d,e^	2.74 ± 0.29 ^c,d^	2.76 ± 0.28 ^b,c^

Values are means ± SEM, n = 20 per treatment group. Means in a row without a common superscript letter differ (*p* ≤ 0.05) as analyzed by one-way ANOVA and Duncan’s multiple range test.

**Table 4:** Effects of different concentrations of auxins, i.e., 1-naphthaleneacetic acid (NAA), indole-3-acetic acid (IAA), and indole 3-butyric acid (IBA), on root formation from regenerated shoots of *R*. *chalepensis* after 6 weeks of incubation.

Treatments	Response%	Number of Roots/Microshoots	Root Length (cm)
¼ MS	57.24 ± 0.39 ^d^	2.62 ± 0.74 ^b^	2.48 ± 0.35 ^b^
½ MS	91.63 ± 2.88 ^a^	5.48 ± 0.51 ^a^	4.91 ± 0.39 ^a^
¾ MS	71.80 ± 0.47 ^b^	3.44 ± 0.51 ^b^	2.66 ± 0.55 ^b^
1 MS	66.24 ± 0.65 ^c^	2.81 ± 0.58 ^b^	2.56 ± 0.16 ^b^

Values are means ± SEM, *n* = 20 per treatment group. Means in a row without a common superscript letter differ (*p* ≤ 0.05) as analyzed by one-way ANOVA and Duncan’s multiple range test.

**Table 5:** RAPD primers used to the genetic fidelity of *R*. *chalepensis* plantlets.

Name of Primers	Sequence 5′–3′	Ta (°C)	No. of Bands
GL A-01	CAGGCCCTTC	33.6	13
GL A-02	TGCCGAGCTG	33.6	3
GL A-03	AGTCAGCCAC	29.5	14
GL A-04	AATCGGGCTG	29.5	11
GL A-05	AGGGGTCTTG	29.5	9
GL A-06	GGTCCCTGAC	33.6	4
GL A-07	GAAACGGGTG	29.5	13
GL A-08	GTGACGTAGG	29.5	9
GL A-09	GGGTAACGCC	33.6	6
GL A-10	GTGATCGCAG	29.5	15
GL B-01	GTTTCGCTCC	29.5	8
GL B-02	TGATCCCTGG	29.5	2
Total no. of bands			107
Average no. of bands/primers			8.92

Ta: annealing temperature.

**Table 6:** DAMD primers used to assess the genetic fidelity *R*. *chalepensis* plantlets.

Name of Primers	Sequence 5′–3′	Ta (°C)	No. of Bands
HVR	GCTCCTCCCCTCCT	50	10
HBV3	GGTGAAGCACAGGTG	53	12
HBV5	GGTGTAGAGAGGGGT	56	15
M13	GAGGGTGGCGGTTCT	57	10
33.6	GGAGGTGGGCA	52	14
Total number of bands			61
Average no. of bands/primers			12.2

Ta: annealing temperature.

## Data Availability

Data is contained within the article.
